# Learning to diagnose accurately through virtual patients: do reflection phases have an added benefit?

**DOI:** 10.1186/s12909-021-02937-9

**Published:** 2021-10-07

**Authors:** Maximilian C. Fink, Nicole Heitzmann, Matthias Siebeck, Frank Fischer, Martin R. Fischer

**Affiliations:** 1 Institute of Medical Education, University Hospital, LMU Munich, Pettenkoferstr. 8a, 80336 Munich, Germany; 2 Institute of Education, Universität der Bundeswehr München, Neubiberg, Germany; 3 Department of Psychology, LMU Munich, Munich, Germany; 4 Munich Center of the Learning Sciences, LMU Munich, Munich, Germany

**Keywords:** Reflection Phases, Diagnostic Competences, Simulation, Medical Education

## Abstract

**Background:**

Simulation-based learning with virtual patients is a highly effective method that could potentially be further enhanced by including reflection phases. The effectiveness of reflection phases for learning to diagnose has mainly been demonstrated for problem-centered instruction with text-based cases, not for simulation-based learning. To close this research gap, we conducted a study on learning history-taking using virtual patients. In this study, we examined the added benefit of including reflection phases on learning to diagnose accurately, the associations between knowledge and learning, and the diagnostic process.

**Methods:**

A sample of *N* = 121 medical students completed a three-group experiment with a control group and pre- and posttests. The pretest consisted of a conceptual and strategic knowledge test and virtual patients to be diagnosed. In the learning phase, two intervention groups worked with virtual patients and completed different types of reflection phases, while the control group learned with virtual patients but without reflection phases. The posttest again involved virtual patients. For all virtual patients, diagnostic accuracy was assessed as the primary outcome. Current hypotheses were tracked during reflection phases and in simulation-based learning to measure diagnostic process.

**Results:**

Regarding the added benefit of reflection phases, an ANCOVA controlling for pretest performance found no difference in diagnostic accuracy at posttest between the three conditions, *F*(2, 114) = 0.93, *p* = .398. Concerning knowledge and learning, both pretest conceptual knowledge and strategic knowledge were not associated with learning to diagnose accurately through reflection phases. Learners’ diagnostic process improved during simulation-based learning and the reflection phases.

**Conclusions:**

Reflection phases did not have an added benefit for learning to diagnose accurately in virtual patients. This finding indicates that reflection phases may not be as effective in simulation-based learning as in problem-centered instruction with text-based cases and can be explained with two contextual differences. First, information processing in simulation-based learning uses the verbal channel and the visual channel, while text-based learning only draws on the verbal channel. Second, in simulation-based learning, serial cue cases are used to gather information step-wise, whereas, in text-based learning, whole cases are used that present all data at once.

**Supplementary Information:**

The online version contains supplementary material available at 10.1186/s12909-021-02937-9.

## Introduction

A recent meta-analysis revealed that simulation-based learning has a large positive effect on learning complex skills, including diagnostic competences in medicine [1]. Moreover, there is evidence that the positive effects of simulation-based learning may be enhanced by combining it with instructional support measures [2–4]. Indeed, numerous studies have confirmed that reflection phases are a particularly effective type of instructional support [5–7]. However, a closer inspection of these studies shows that reflection phases were primarily investigated for text-based cases and not for simulation-based learning with virtual patients. Therefore, to what extent reflection phases can foster learning to diagnose accurately in simulation-based learning is an open question. . Below, we summarize our study’s underlying conceptual framework, define virtual patients and text-based cases, and discuss the potential effect of reflection on facilitating diagnostic competences.

### Underlying conceptual framework

Our study is based on the conceptual framework for acquiring diagnostic competences in simulations with instructional support by Heitzmann et al. [8]. They define simulations as models of diagnostic situations that can be manipulated and sometimes even controlled by participants. The instructional support provided can include, for instance, examples, prompts, or reflection phases. The effectiveness of simulation-based learning with instructional support depends on individuals’ diagnostic process and prerequisites such as prior knowledge. The diagnostic process can be operationalized through eight diagnostic activities, including the *current hypothesis* (preliminary diagnosis) learners form in the course of diagnosing. Knowledge encompasses the two types *conceptual knowledge* and *strategic knowledge*. Conceptual knowledge refers to knowledge about constructs and their relations, while strategic knowledge is defined as knowledge about heuristics and strategies in diagnosing. The primary outcome measure of simulation-based learning in this framework is *diagnostic accuracy* - the agreement between the participant’s diagnosis and a correct sample solution [8]. Next, we will define virtual patients and text-based cases and briefly describe possible differences in information processing while learning from them.

### Virtual patients and text-based cases

*Virtual patients* are a special type of computer simulation representing clinical situations such as history-taking or physical examinations [9]. Moreover, virtual patients frequently include audio-visual material as well as text-based information [10, 11]. The *text-based cases* used in studies on reflection phases typically consist of a description of the patient’s main symptoms, as well as relevant findings from history-taking, the physical examination, and lab investigations [5–7]. Two theoretical perspectives suggest that information processing during learning from virtual patients and text-based cases may differ. According to the cognitive theory of multimedia learning [12], humans possess two separate channels for visual and verbal information processing that are used during learning [13]. Consequently, learners will process virtual patients using both channels, while text-based cases will only be processed in the channel for verbal information. Moreover, differences in the case formats could determine how information is processed [11, 14]. Virtual patients typically represent *serial cue* cases, in which information is obtained step-wise by navigating through a digital environment. In text-based cases, information is typically presented in the *whole case* format, in which all relevant information is displayed at once. In the following section, we will discuss the potential effect of reflection on facilitating diagnostic competences.

### Reflection and facilitating diagnostic competences

Reflection is defined as a cognitive and metacognitive process in which learners deal with their thoughts and actions, as well as their bases, intending to modify them [15]. On the one hand, reflective processes can implicitly occur in virtual patients containing design features that provide opportunities for this. On the other hand, *reflection phases* as instructional support can explicitly induce beneficial reflective processes by providing specific instructions and a dedicated phase of time for this activity. In medical diagnosing interventions, the instructions for reflection phases typically include questions on the initial hypothesis, alternative hypotheses, and reasons for and against these hypotheses [5, 7, 16]. The effectiveness of reflection has been primarily tied to dual-process theory [17], which claims that two cognitive systems are used in diagnosing: a fast, heuristic, and a slow, reflective system. In line with Mamede et al. [18], reflection phases induce slow cognitive processes that could be particularly beneficial for correcting mistakes caused by faulty heuristic diagnosing. Current research on reflection phases centers around (1) the effectiveness of reflection, (2) the associations between prior knowledge and learning from reflection, and (3) the quality of the diagnostic process.

Concerning (1) the effectiveness of reflection, a meta-analysis on instructional support in problem-centered instruction in the domains of medical education and teacher education reported a medium positive effect (*g* = 0.58) of including reflection phases on promoting diagnostic competences [2]. In addition, a literature review for medical education by Mamede et al. [18] found that reflection phases facilitated diagnostic competences in most studies that used them to validate diagnoses with specific reasoning instructions. At this point, it should be noted that the medical education literature primarily investigated the effect of reflection phases for learning from text-based cases, while results for simulation-based learning are lacking. In contrast, a cross-domain meta-analysis focused on simulation-based learning discovered no added benefit of including reflection phases on fostering complex skills [1]. In sum, there is more evidence that reflection phases are effective than not effective at fostering diagnostic competences in medicine. Despite opposing findings from other domains, we currently assume that this is also true in the context of simulations.

The (2) associations between prior knowledge and learning from reflection should also be examined. Support for this association comes from the two aforementioned meta-analyses on instructional support in problem-centered instruction and simulation-based learning [1, 2], which both showed that reflection phases were more beneficial for college students with high prior knowledge than with low prior knowledge. In these meta-analyses, learners’ prior knowledge was measured dichotomously (low vs. high) based on years of academic training and content familiarity. In partial contrast to these results, an experiment by Mamede et al. [19] demonstrated that physicians in specialty training but not undergraduate medical students benefitted from conscious, slow diagnostic thinking when solving complex problems. The authors argued that the undergraduate college students in their study did not possess the necessary knowledge foundation to experience improvement through reflective processes. In short, the literature indicates that learners with higher prior knowledge benefit more from reflection phases than learners with lower prior knowledge. However, further research on the level of expertise required to profit from reflection phases is necessary.

Two topics concerning the quality of the diagnostic process (3) should be investigated further. First, the diagnostic process during reflection phases should be examined by inspecting learners’ hypotheses. Mamede et al. [20] showed that hypotheses improved from a first point in the diagnostic process before reflection to a second point in the diagnostic process after reflection. In their study, four different types of reflection phases (no specific instructions, arguments for the diagnosis, arguments against the diagnosis, and arguments for and against the diagnosis) were applied to text-based cases. As is the case during reflection phases, learners might also be able to enhance their hypotheses over the course of simulation-based learning without reflection phases by gathering and interpreting additional data [8]. Second, the optimal timing of reflection phases within the diagnostic process should be analyzed. Initial evidence highlights that reflection phases are particularly effective *during* rather than before or after diagnosing [18]. However, two different operationalizations of reflection phases during diagnosing are conceivable: In *accompanying reflection*, learners reflect in the middle of a case and then continue working on it before providing a final diagnosis. In *concluding reflection*, learners reflect after completing a case, right before providing a final diagnosis. Each type of reflection phases could have specific benefits. Accompanying reflection could primarily help learners plan and monitor their ongoing diagnostic process in the sense of improved self-regulated learning [21]. Concluding reflection could offer learners more case information to reconsider in the sense of self-generated feedback to be used in problem-solving [22]. In light of the potential benefits of accompanying reflection over concluding reflection for the diagnostic process, we assume that this type of instructional support is particularly effective for virtual patients with serial cue cases.

### Research questions and hypotheses

To investigate reflection phases in the context of simulations, we address the following research questions: To what extent do reflection phases affect learning to diagnose accurately in virtual patients? (RQ1) We hypothesize that the inclusion of reflection phases in simulation-based learning has an added benefit for learning to diagnose accurately (H1.1). Furthermore, we assume that accompanying reflection is more beneficial for learning to diagnose accurately than concluding reflection (H1.2). To what extent is prior knowledge associated with learning to diagnose accurately through reflection phases? (RQ2) We expect that learners with higher conceptual (H2.1) and strategic (H2.2) knowledge would experience greater improvement in diagnostic accuracy than learners with lower prior knowledge of these types. To what extent does the diagnostic process improve during simulation-based learning with virtual patients and during reflection phases, in the sense of enhancements in current hypotheses and diagnostic accuracy over the course of cases? (RQ3) We assume that the diagnostic process improves both during simulation-based learning (H3.1) and reflection phases (H3.2).

## Method

### Sampling procedure, participants, and research design

Data collection for the study ran from October 2019 to February 2021. Recruitment took place on-campus and through online advertising. Medical students from LMU Munich with high German language proficiency in their third to fifth year of medical school were eligible. The final sample consisted of *N* = 121 participants with an average age of *M* = 24.90 years, *SD* = 4.01 years. The gender of participants was distributed as follows: *n* = 82 (67.7 %) female, *n* = 10 (8.3 %) male, and *n* = 29 (24.0 %) no answer. The high proportion of participants with no answer on gender was likely caused by the use of an electronic form that allowed skipping this question without selecting an option. The final sample represents about 5 % of the enrolled third to fifth year medical students from LMU Munich and is representative in age for this population. We report more details on the sampling and participants in Additional file 1: Appendix S1 and S2.

The study used a pretest-posttest design, varying the type of reflection. Participants were randomly assigned to one of three conditions: (1) concluding reflection (*n* = 42), (2) accompanying reflection (*n* = 39), and (3) control group (*n* = 40). Data collection moved from the lab to the web in the middle of the study due to the COVID-19 pandemic. In both types of data collection, an identical learning environment was used. In lab-based data collection, an experimenter was present in the computer room at the university hospital. In web-based data collection, an experimenter was connected via video chat. The proportion of participants experiencing each data collection method across conditions are provided in Additional file 1: Appendix S2. A chi-square test showed that the proportions participants experiencing each data collection method did not differ across the conditions, $\chi$²(2, *N* = 121) = 0.01, *p* = .994.

### Procedure

**Fig. 1:**
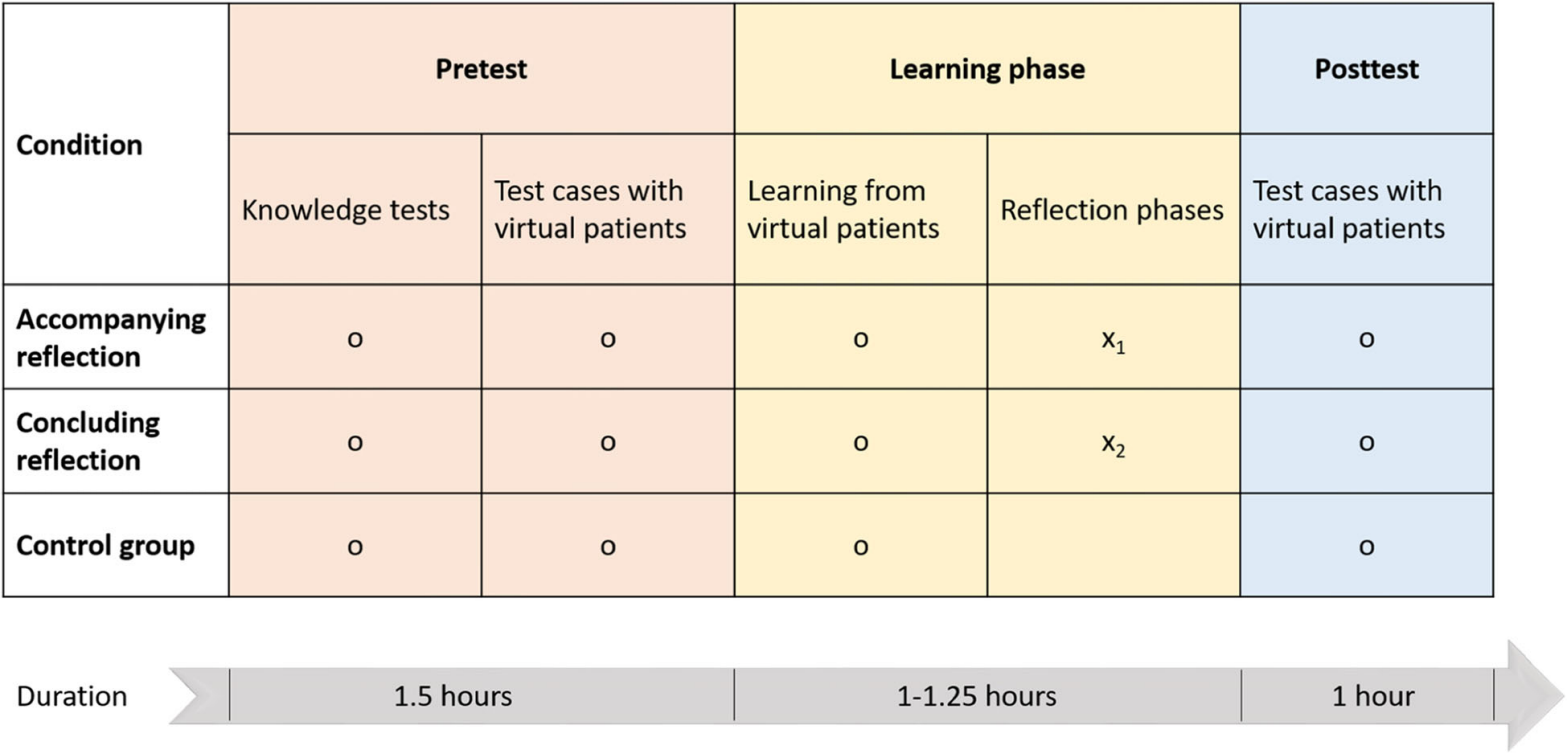
Illustration of the study procedure, including approximate durations. Note on the symbols: o Indicates a measurement, x Indicates a treatment. Details on the intervention: X_1_: Reflection halfway through each case, X_2_: Reflection after completing each case

We provide a visualization of the procedure for the different conditions in Fig. 1. Participants began the pretest by completing the conceptual and strategic knowledge tests to assess their prior knowledge. The conceptual and strategic knowledge tests are described in more detail later. Next, participants completed a familiarization with the simulation-based learning environment and then diagnosed three virtual pretest patients. During the learning phase, all participants solved three other virtual patients. In all conditions, participants were reminded via prompts to spend a minimum of 5 min on each simulation and had to stop working on the simulation after a time limit of 10 min. We selected the time limit of 10 min based on a prior study using similar cases [23]. Our goal was to provide sufficient time for diagnosing with an efficiency mindset but without inducing severe time pressure. In the accompanying reflection condition, a reflection phase took place halfway through each case. In the concluding reflection condition, a reflection phase was conducted after completing each case but before providing a final diagnosis. Moreover, only during the learning phase and in all conditions, including the control group, a video-based expert solution was presented after fully completing and diagnosing each virtual patient. The expert solution contained the correct diagnosis and strategic knowledge on the correct diagnostic process. In the posttest, participants completed three additional virtual patients.

### Materials

#### Virtual patients

**Fig. 2:**
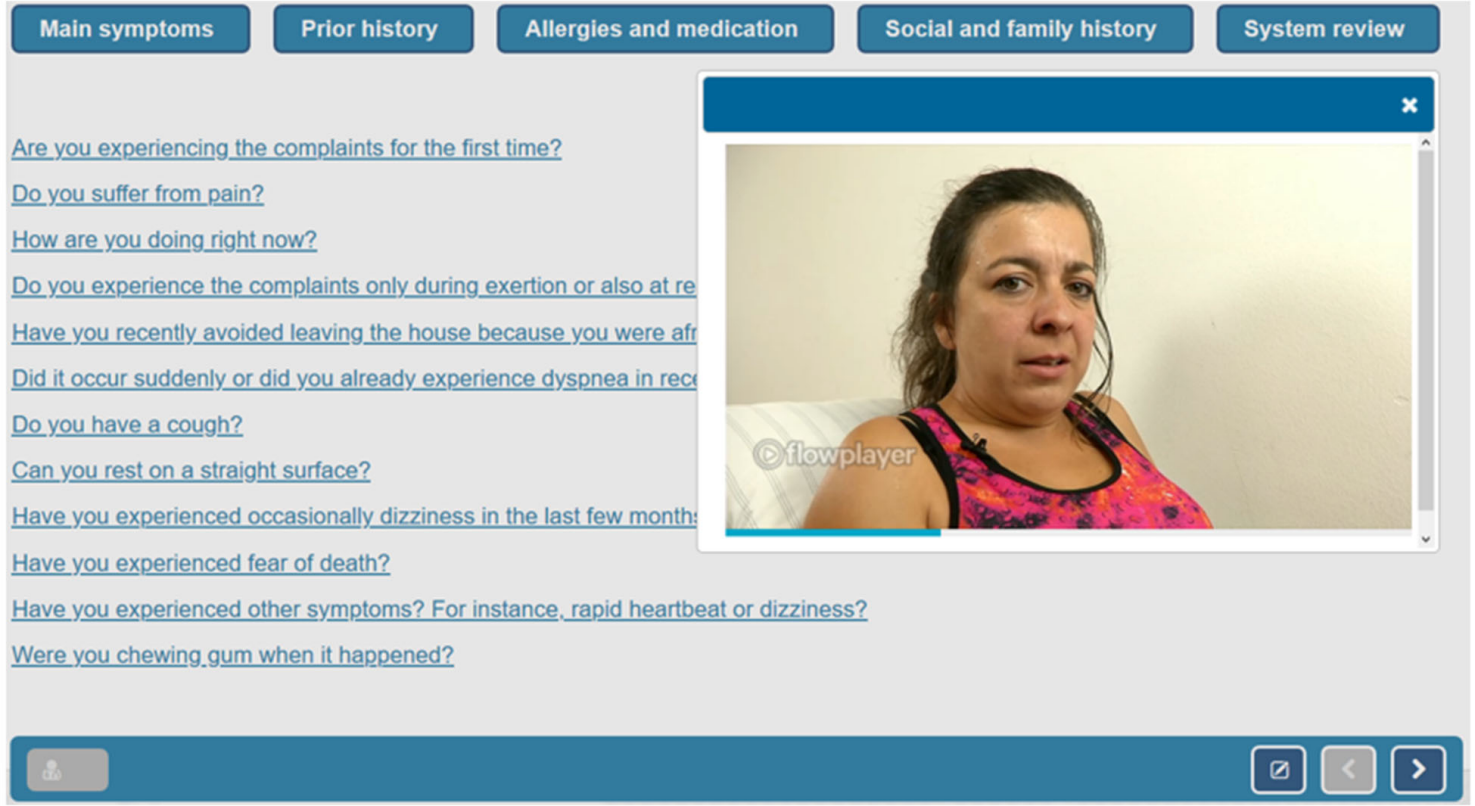
Virtual patient by Fink et al. [23] licensed under CC BY 4.0

Participants diagnosed nine virtual patients suffering from different causes of dyspnea. The virtual patients were validated in a study by Fink et al. [23]. In Additional file 1: Appendix S3, we provide an overview of the diagnoses and characteristics of the virtual patients. The virtual patients of the learning phase were selected so that a transfer to the virtual patients of the pre- and posttest was possible. In fact, the learning phase contained various cardiopulmonary perfusion and diffusion problems that shared a common hypothesis space with the pre- and posttest. The (semi)-professional actors playing the patients were selected based on the virtual patients’ characteristics and trained for their role by an acting coach and a physician. The created virtual patients were then embedded into the digital learning environment CASUS [24]. We present a screenshot of one of the virtual patients in Fig. 2.

At the beginning of each virtual patient encounter, prior diagnostic information (e.g., lab results) and the chief complaint were presented in an introductory video. Then, participants took the patient´s history by selecting from a menu of 69 questions (cf. the questions on the left of Fig. 2). The answer to each selected history-taking question was streamed as a short video. Additional file 1: Appendix S3 provides examples of the history-taking questions used and a source for the complete list of history-taking questions.

#### Reflection phases

The content for the accompanying and concluding reflection conditions were based on scripts developed by Mamede et al. [5, 7, 16]. As previously mentioned, in *accompanying reflection*, learners reflected after 5 min equaling halfway through working on a case. In *concluding reflection*, learners reflected after completing the case, right before offering their final diagnosis. The scripts for both types of reflection consisted of nine questions and are documented in Additional file 1: Appendix S4. Participants received 4 min and 20 s within each case to engage in reflection.

### Instruments

#### Diagnostic accuracy

Diagnostic accuracy was measured in each virtual patient with a long menu consisting of 180 possible diagnoses related to dyspnea. Participants selected one diagnosis per case, which was compared to a solution. One point was awarded for the designated correct answer, 0.50 points for a partially correct answer, and 0 points for all other diagnoses. The learners’ answers were compared using *R* scripts to the common sample solution of two expert physicians validated in Fink et al. [23]. Mean scores for diagnostic accuracy were calculated for the pretest, posttest, and the learning phase and ranged from 0 (*low*) to 1 (*high*). The third case in the pretest (diagnostic accuracy *M* = 0.05, *SD* = 0.14) and the second case in the posttest (*M* = 0.08, *SD* = 0.23) were excluded from our analyses because of floor effects (see Additional file 1: Appendix S3 for the diagnoses in these cases).

#### Current hypothesis in the diagnostic process

To assess participants’ current hypothesis in the diagnostic process, we proceeded as follows. We asked participants in every condition to select their current hypothesis for each patient from the same long menu described for diagnostic accuracy directly after reading the prior diagnostic information and watching the chief complaint on video. Moreover, participants’ current hypothesis was additionally measured at the start and the end of each type of reflection.

#### Conceptual knowledge test

The conceptual knowledge tests focused on dyspnea and history-taking. The test consisted of 20 items and contained single-choice and pick-N multiple-choice questions. In single-choice questions, participants received one point for the correct answer. In pick-N multiple-choice questions, participants received one point if their entire answer pattern was correct. If participants selected more than 50 % correct answers in a pick-N multiple-choice question, they were awarded 0.50 points, in line with Bauer et al. [25]. Conceptual knowledge scores were determined by dividing the number of points achieved by the number of questions posed. Thus, conceptual knowledge scores ranged from 0 (*low knowledge*) to 1 (*high knowledge*). The time limit for the test was set to 20 min. The reliability was acceptable, with Cronbach´s $\alpha$ = 0.61.

#### Strategic knowledge test

Strategic knowledge on dyspnea and history-taking was assessed with four key feature cases [26]. Each case consisted of four single-choice questions regarding the diagnosis, treatment, symptoms, and further diagnostic measures. One point was awarded for each correct answer. Strategic knowledge test scores were calculated by dividing the number of points achieved by the number of questions posed. Therefore, strategic knowledge scores ranged from 0 (*low knowledge*) to 1 (*high knowledge*). Testing time was set to 20 min. The test’s reliability was acceptable, with Cronbach´s $\alpha$ = 0.65.

#### Cognitive load

Cognitive load was assessed as a control variable once directly after the end of the learning phase. We measured this variable as a control variable because a negative association between cognitive load and performance in medical skills, such as diagnosing, has been shown repeatedly [27]. Moreover, reflection phases could affect the cognitive load present in the different experimental conditions. We used for the assessment of cognitive load a five-item, five-point scale by Opfermann [28]. The scale differentiates between germane, extraneous, and intrinsic cognitive load and lets participants rate their mental effort from (1) very low to (5) very high.

### Manipulation checks

One manipulation check on duration showed that, as intended, participants in the intervention groups spent about four additional minutes on the reflection phase for each case (see Additional file 1: Appendix S5). We consider this sufficient time for reflection in cases with a time limit of 10 min. Another manipulation check confirmed that participants successfully engaged in reflection by writing a sufficient amount of notes in our digital environment (see Additional file 1: Appendix S5).

### Statistical analyses and sample size

We used R (Version 4.0.2) [29] for the statistical analyses. We investigated RQ1 with an analysis of covariance. RQ2 was examined with one-tailed Pearson correlations. For RQ3, we used one-tailed paired sample t-tests. In all statistical analyses, the significance level was set to $\alpha$ = 0.05.

An a priori-power analysis was conducted with G*Power (Version 3.1) [30], assuming an error probability of $\alpha$ = 0.05 and a power of $\beta$ = 0.80. For the main analysis of RQ1, we hypothesized that the effect of reflection phases on learning to diagnose accurately would be medium-sized, with *g* = 0.58, based on the meta-analysis by Chernikova et al. [2]. Based on this assumed effect size, the power analysis yielded a required sample size of *N* = 118 participants with 39 participants per group.

## Results

### Preliminary analyses

We report descriptive statistics and results from a one-way analysis of variance for knowledge, diagnostic accuracy, and cognitive load in Table 1. These results show that knowledge and diagnostic accuracy did not differ across the experimental conditions in the different phases of the experiment. Similarly, cognitive load control variables did not differ across the experimental conditions when they were measured directly after the learning phase.

**Table 1:** Descriptive statistics and ANOVA results for knowledge, diagnostic accuracy, and cognitive load

	Concluding reflection	Accompanying reflection	Control group	*F*	*df*	*p*
Conceptual knowledge - pretest	0.52 (0.13)	0.57 (0.14)	0.53 (0.13)	1.80	2, 115	0.169
Strategic knowledge - pretest	0.50 (0.14)	0.50 (0.15)	0.50 (0.13)	0.03	2, 108	0.973
Diagnostic accuracy in VPs - pretest	0.45 (0.31)	0.49 (0.34)	0.47 (0.28)	0.24	2, 115	0.785
Diagnostic accuracy in VPs - learning phase	0.56 (0.25)	0.59 (0.16)	0.55 (0.19)	0.46	2, 117	0.630
Diagnostic accuracy in VPs - posttest	0.44 (0.33)	0.37 (0.31)	0.34 (0.33)	1.03	2, 117	0.360
Cognitive load control variables						
Extraneous CL	2.96 (0.88)	2.81 (0.68)	2.77 (0.73)	0.71	2, 118	0.493
Intrinsic CL	3.31 (0.95)	3.18 (0.79)	3.30 (0.85)	0.28	2, 118	0.759
Germane CL	2.55 (0.97)	2.46 (0.79)	2.52 (0.82)	0.11	2, 118	0.899

*Note.* Descriptive statistics and results of one-way ANOVAs for knowledge, diagnostic accuracy, and cognitive load across the three experimental conditions. Diagnostic accuracy and knowledge ranged from (0) *entirely incorrect* to (1) *entirely correct*. In concluding reflection, reflection phases took place after completing each case. In accompanying reflection, reflection phases took place halfway through each case. In the control group, no reflection phases were provided. Cognitive load variables were measured once directly after the learning phase and ranged from (1) *very low* to (5) *very high*

*VPs *virtual patients, *CL* cognitive load

### The effect of reflection phases on learning to diagnose accurately (RQ1)

To answer RQ1, we conducted an analysis of covariance using the diagnostic accuracy score from the posttest as the outcome. After adjustment for pretest diagnostic accuracy, there was no statistically significant difference in posttest diagnostic accuracy between the conditions, *F*(2, 114) = 0.93, *p* = .398, $\eta$*p*^2^ = 0.02. Thus, H1.1, an added benefit of reflection phases on learning to diagnose accurately, could not be confirmed. A pairwise comparison showed that, in contrast to H1.2, accompanying reflection and concluding reflection did not differ from each other, *t*(114) = 0.93, *p* = .356.

### The association between prior knowledge and learning to diagnose accurately through reflection phases (RQ2)

Next, we examined whether prior knowledge and learning to diagnose accurately through reflection phases were associated. Across both reflection groups, the gain in diagnostic accuracy from pretest to posttest was not correlated with either pretest conceptual knowledge (*r* = .12, *p* = .139) or strategic knowledge (*r* = .10, *p* = .207). Therefore, H2.1 and H2.2 were not substantiated. A follow-up analysis on the correspondence between both types of prior knowledge showed that there was a medium correlation between conceptual and strategic knowledge (*r* = .55, *p* < .001).

### Improvement in the diagnostic process during simulation-based learning and in reflection phases (RQ3)

Finally, we investigated the extent to which participants’ diagnostic process improved during simulation-based learning and in reflection phases. To do so, we examined the scores for current hypothesis and diagnostic accuracy, which used the same long menu that included 180 possible diagnoses related to dyspnea. Detailed descriptive statistics for our analyses are presented in Table 2.

**Table 2:** Descriptive statistics for the diagnostic process during the learning phase

	Current hypothesis – start of VPs	Current hypothesis – start of reflection phase	Current hypothesis – end of reflection phase	Diagnostic accuracy - end of VPs	
Control group	0.45 (0.21)	-	-	0.55 (0.19)	
Reflection conditions	0.39 (0.21)	0.50 (0.21)	0.57 (0.25)	0.58 (0.21)	

*Note.* Current hypothesis and diagnostic accuracy were measured as indicators of the diagnostic process several times during the experiment. Both variables were assessed with the same instrument and ranged from (0) *entirely incorrect* to (1) *entirely correct*. Means and SDs are provided in the table above separately for the control group and both reflection conditions (accompanying and concluding reflection) combined. For the control group, the current hypothesis at the start of the VPs and diagnostic accuracy at the end of the VPs are given. In the reflection conditions, the current hypothesis at the start and the end of the reflection phases is also reported

*VPs* virtual patients

For simulation-based learning without reflection phases (the control group), a paired samples t-test demonstrated that participants’ diagnostic accuracy after working with the virtual patients was significantly higher than their current hypothesis at the start of the virtual patient encounters (*t*(39) = 3.08, *p* = .002). This finding corroborates H3.1, that participants’ diagnostic process improves during simulation-based learning. A follow-up categorical analysis of the learning process showed that not changing one’s hypothesis (71.6 %) was more frequent than improvement (22.0 %) or deterioration (6.4 %). Looking closer at the category of not changing one’s hypothesis in this analysis, 28.4 % participants adhered to a fully correct hypothesis, 22.9 % stuck with a partially correct hypothesis, and 20.2 % kept an incorrect hypothesis^1^.

Changes in current hypothesis over the reflection phases were investigated for both reflection conditions combined. A paired samples t-test showed that participants improved their current hypothesis from the start to the end of reflection phases (*t*(73) = 2.73, *p* = .004). This result substantiates H3.2, that participants enhance their diagnostic process in reflection phases. Examining this part of the learning process categorically, not changing one’s hypothesis (90.1 %) was more frequent than improvement (7.0 %) or deterioration (2.9 %). Focusing on the category of not changing one’s hypothesis in the last analysis, 32.2 % of the participants adhered to a fully correct hypothesis, 32.7 % stuck with a partially correct hypothesis, and 25.1 % kept an incorrect hypothesis^2^. Moreover, an explorative paired samples t-test of the reflection conditions showed that the participants’ diagnostic accuracy at the end of the virtual patient encounter was significantly higher than their current hypothesis at the start of the virtual patient encounter (*t*(79) = 7.91, *p* < .001). Analyzing this part of the learning process categorically, not changing one’s hypothesis (66.6 %), was more frequent than improvement (29.7 %) and deterioration (3.7 %).² Inspecting the category of not changing one’s hypothesis for this analysis closer, 24.2 % of the participants adhered to a fully correct hypothesis, 21.5 % stuck with a partially correct hypothesis, and 21.0 % kept an incorrect hypothesis.

## Discussion

### Principal findings

Regarding the first research question (RQ1), we observed no added benefit of reflection phases for learning to diagnose accurately. This finding is not in line with the medium effects of reflection phases and other instructional supports on cognitive outcomes in problem-centered instruction [2, 4]. However, our finding corresponds to new meta-analytic results that reflection has no additional benefit for complex skills in simulation-based learning [1]. 

One difference between simulation-based learning and problem-centered instruction that could explain the differential effects is their average effectiveness. Simulation-based learning has a large effect on learning [1], while the effect of problem-centered instruction is moderate [2, 4]. Consequently, adding reflection to simulation-based learning might not lead to a further increase in the highly beneficial effect of simulation-based learning itself. This explanation, however, is not supported by the fact that other instructional supports and particular combinations of instructional supports demonstrated added benefits in simulation-based learning [1]. 

Another difference between simulation-based learning and problem-centered instruction that could influence reflection phases’ effectiveness could be cognitive load. However, our control analysis on cognitive load showed that cognitive load in the virtual patients reached medium values comparable to problem-centered instruction with text-based cases [14]. Our results can be compared to the results for the text-based cases because exactly the same cognitive load scale was used in these two studies. Therefore, we can infer that cognitive load was not excessively high in our virtual patients. Moreover, cognitive load did not differ across the experimental conditions, suggesting that reflection phases did not manipulate cognitive load. 

A more plausible explanation for the discovered differential effectiveness of reflection phases in simulation-based learning and problem-centered instruction concerns the case format. In simulation-based learning, serial cue cases are typically utilized, which was also true in our experiment. Serial cue cases present data in a step-wise fashion and involve interactive case construction and interpretation [11, 14]. In problem-centered instruction, text-based whole cases are typically used. Whole cases require the learner to remember and interpret all of the information that is presented [11, 14]. Comparing both case formats, it can also be argued, that serial cue cases may perhaps provide by their very nature more room for implicit reflective processes than whole cases. The lack of effect of reflection phases in our study could be explained by the differences between these case formats as follows. Reflection phases might be less effective in serial cue cases when cases are interactively constructed, and there is room for implicit reflective processes. However, reflection phases might be more effective in whole cases when interpreting the full case information is essential, and there is little room for implicit reflective processes. 

Another plausible explanation for the difference in the effectiveness of reflection phases in simulation-based learning and problem-centered instruction is based on the theory of multimedia learning [12]. According to this theory, information processing differs during simulation-based learning and problem-centered instruction using text-based cases. The finding that reflection phases had no effect on learning to diagnose accurately in our study but are generally effective in problem-centered instruction can be explained according to this theory as follows. In simulation-based learning with virtual patients, the visual and the verbal channels are used simultaneously, and the largest benefit for learning may arise from integrating both channels [31]. Reflection phases might not support this integration process. In problem-centered instruction based on text cases, however, only the verbal channel is used. Reflection phases might particularly support the cognitive processes of selecting and organizing words that are important for creating an elaborate verbal representation [31]. 

Moreover, to complement our main research question, we examined the optimal timing of reflection phases. We initially assumed that accompanying reflection would outperform concluding reflection due to improved planning and monitoring of the diagnostic process [21]. Nevertheless, we also acknowledged that the concluding reflection condition might be associated with creating better self-generated feedback to be used in problem-solving [22]. However, the two reflection conditions had no effect on learning to diagnose accurately and did not differ from each other. Our findings suggest that in simulation-based learning, the two types of reflection phases do not differ in their effectiveness, and none of the described mechanisms is highly beneficial.

In the second research question (RQ2), we examined the associations between prior knowledge and learning to diagnose accurately through reflection phases. Neither conceptual nor strategic prior knowledge was correlated with improvements in diagnostic accuracy through reflection phases. This finding contradicts results from meta-analyses that learners with high prior knowledge benefit more from reflection than learners with low prior knowledge [1, 2]. However, there is a convincing explanation for this finding. In the described meta-analyses, knowledge was mainly operationalized as expertise determined by years of training. From an expertise development perspective, we investigated third to fifth year undergraduate medical students in our study, a cohort of learners with low to medium expertise. This cohort of learners was not able to learn through reflection phases in the context of virtual patients. This finding corresponds to an experiment by Mamede et al. [19], which showed that only postgraduate students and not undergraduate students, benefited from conscious, slow thinking when solving complex text-based cases. Together, our study and, even more convincingly, the experiment by Mamede et al. [19] indicate that reflection phases’ effectiveness for learning to diagnose accurately might depend more on large differences in expertise than on smaller, context-specific differences in knowledge.

In the third research question (RQ3), we analyzed the extent to which participants’ diagnostic process improves during simulation-based learning and reflection phases. 

It is important to note that the improvements in the diagnostic process we reported probably depend to some extent on case difficulty. On the one hand, greater improvements during simulation-based learning and reflection phases are possible with more difficult cases. On the other hand, improvements are presumably impossible with overly difficult cases. The separately reported proportions of not changing one’s hypothesis (specifying the proportion of fully correct, partially correct, and incorrect unchanged hypotheses), improvement, and deterioration suggest sufficient room for improvement during virtual patients and reflection phases. 

The analysis of the simulation-based learning phase without reflection phases (the control group) demonstrated that participants improved their diagnoses from the start of each case to the end. A categorical follow-up analysis showed that a substantial number of participants improved. This improvement in the diagnostic process might, on the one hand, be explained by the step-wise gathering and interpretation of additional data while working with the virtual patients [8]. On the other hand, the expert sample solutions provided after participants gave their final diagnosis in each case might have also had a positive transfer effect on participants’ diagnoses in the subsequent virtual patients. 

The analysis of the diagnostic process during the reflection phases (both intervention groups) revealed that participants also improved their current hypotheses from the start of the reflection phase to the end. A categorical follow-up analysis showed that a smaller proportion of participants improved their current hypotheses during the reflection phases than while working with the virtual patients. 

Together, these findings indicate that simulation-based learning with the virtual patients contributed more substantially to participants’ improvements in the diagnostic process than reflection phases. Furthermore, improvements in the diagnostic process during the virtual patients and in the reflection phases we discovered in the learning phase did not transfer to an improved diagnostic accuracy in the posttest. There are two explanations we suggest for this finding. First, the reflection phases might not have been as effective as expected due to differences in case format and information processing (please see discussion for RQ1). Second, the expert solutions we included in all three experimental conditions during the learning phase could have affected posttest performance concerning diagnostic accuracy more strongly than the reflection phases [32]. More specifically, the expert solutions included strategic knowledge on the correct diagnostic process that may have contributed to reducing the differences between the control and reflection groups. However, providing feedback in the form of expert solutions is frequently considered a necessary part of simulation-based learning [33]. Therefore, we argue that it made sense to include expert solutions in all conditions. 

To link our findings more closely to other research, we would like to briefly highlight similarities and differences between debriefing and the expert solutions and reflection phases used in our study. Debriefing can stimulate reflection processes and include solutions to the diagnostic process or performance [34]. In contrast to reflection phases and expert solutions, however, debriefing is more interactive and dialogic [34]. Thus, our findings cannot be generalized to debriefing, for which much further research seems necessary and valuable.

In conclusion, instructional support in the form of reflection phases had no added benefit for learning to diagnose accurately for undergraduate students with low to medium expertise in simulation-based learning with virtual patients. If our findings are replicated, this would suggest that other instructional supports might be more beneficial in this context and similar settings. Combinations of selective instructional support (such as examples and prompts) and adaptive instructional support could be promising alternatives to reflection phases, as both have been found to be beneficial in simulation-based learning and for learners with relatively little expertise [1, 4, 35, 36].

### Limitations

One limitation of the study is that we switched data collection from the lab to the internet in the middle of the data collection period due to the COVID-19 pandemic. The drawback of web-based data collection is that it is considered less controlled than lab-based data collection [37]. However, this limitation should not be considered too severe in this study for two reasons. First, we conducted detailed manipulation checks that showed that the experiment was conducted as intended. Second, the proportions of web-based and lab-based data collection were similar in all conditions, as the Chi-squared test reported in the methods section showed.

Another limitation of the study could be the relatively low number of virtual patient cases we used. Other studies on reflection phases have used a larger number of text-based cases to assess diagnostic competences [6, 16]. The advantages of using a larger number of cases are that case specificity can be mitigated and reliability can be further increased [38, 39]. However, the benefits of using fewer virtual patient cases with a realistic duration, as we did in this study, are that more contextual information is conveyed and participants encounter a more interactive, realistic situation and task with higher validity [40].

A third limitation of the study could be the use of an immediate posttest. Even though positive effects of reflection on diagnostic accuracy have been reported on more immediate measures [20], most studies discovered positive effects on delayed posttests [5–7]. Therefore, it is possible that using a delayed posttest instead of an immediate posttest may have resulted in a positive effect of reflection phases on knowledge organization and retention, which were not assessed in the immediate posttest used.

## Conclusions

We conducted a study on diagnosing in virtual patients with and without reflection phases. Our results showed that reflection phases did not have an added benefit on learning to diagnose accurately. This finding may be limited to the context of virtual patients and undergraduate medical students with low to medium expertise and needs replication. However, the results could have two important implications. First, reflection phases may not be as effective in simulation-based learning as in regular problem-centered instruction using text-based cases. This implication is substantiated by differences in case format and information processing between simulation-based learning and problem-centered instruction with text-based cases. Second, instructional supports other than reflection phases could be more beneficial for medical students with low to medium expertise in the context of simulation-based learning.

## Supplementary Information

**Additional file 1: Appendix S1.** Diagram of participant flow. **Appendix S2.** Participant characteristics. **Appendix S3.** Cases in the virtual patients and history-taking questions. **Appendix S4.** Reflection phases. **Appendix S5.** Manipulation checks.

## Data Availability

The datasets used and analysed during the current study are available from the corresponding author on reasonable request.

## Footnotes

1 The percentages in this analysis differ slightly from the total category percentage due to rounding.

2 The percentages in this analysis differ slightly from the total category percentage due to rounding.
