# Supplementary material for: Learning to diagnose accurately through virtual patients: do reflection phases have an added benefit?
Source: BMC Med Educ. 2021 Oct 7;21:523. doi: 10.1186/s12909-021-02937-9 (PMC8497044; doi:10.1186/s12909-021-02937-9)
Supplement: Supplementary file 1 — Additional file 1: Appendix S1. Diagram of participant flow. Appendix S2. Participant characteristics. Appendix S3. Cases in the virtual patients and history-taking questions. Appendix S4. Reflection phases. Appendix S5. Manipulation checks. [file 12909_2021_2937_MOESM1_ESM.docx]

**APPENDICES**

**Appendix S1: Diagram of participant flow**

Analysed (n=42)
♦ Excluded from analysis (technical difficulties) (n=2 )

Allocated to control group (n=44)

♦ Received allocated intervention (n=44)

♦ Did not receive allocated intervention (give reasons) (n= 0)

Analysed (n=40)
♦ Excluded from analysis (missunderstood instruction, technical difficulties, did not complete study) (n=4)

Assessed for eligibility (n=128)

Randomized (n=128)

Excluded (n=0)

♦  Not meeting inclusion criteria (n=0)

♦  Declined to participate (n=0)

♦  Other reasons (n=0)

Analysed (n=39)
♦ Excluded from analysis (technical difficulties) (n=1)

Allocated to accompanying reflection (n=40)

♦ Received allocated intervention (n=40)

♦ Did not receive allocated intervention (n=0)

Allocated to concluding reflection (n=44)

♦ Received allocated intervention (n=44)

♦ Did not receive allocated intervention (n=0)

## Allocation

## Analysis

## Enrollment

**Appendix S2: Participant characteristics**

*Participant characteristics across all conditions*

|  | Concluding reflection | Accompanying reflection | Control group | All groups |
| --- | --- | --- | --- | --- |
| Age in years mean, (SD) | 25.12 (3.76) | 25.29 (3.90) | 24.30 (4.39) | 24.90 (4.01) |
| Participants | 42 | 39 | 40 | 121 |
| **Sex, n (%)** |  |  |  |  |
| Females | 30 (72) | 28 (72) | 24 (60) | 82 (68) |
| Males | 1 (2) | 3 (8) | 6 (15) | 10 (8) |
| No answer | 11 (26) | 8 (20) | 10 (25) | 29 (24) |
| **Data collection, n (%)** |  |  |  |  |
| Lab-based | 12 (29) | 11 (28) | 11 (27) | 34 (28) |
| Web-based | 30 (71) | 28 (72) | 29 (73) | 87 (72) |

**Appendix S3: Cases in the virtual patients and history-taking questions**

*Cases in the virtual patients*

| Phase of the experiment | Diagnosis | Patient characteristics |
| --- | --- | --- |
| Pretest | Hypertrophic cardiomyopathy | 25 years, male |
|  | Pneumonia | 55 years, female |
|  | Pulmonary embolism in case of prostrate cancer | 70 years, male |
| Learning phase | Acute posterior myocardial infarction | 55 years, female |
|  | Pulmonary embolism due to heparin induced thrombocytopenia | 70 years, male |
|  | Lung cancer | 60 years, female |
| Posttest | Pulmonary embolism due to coagulation disorder | 35 years, female |
|  | Congestive heart failure with atrial fibrillation | 65 years, female |
|  | Hyperventilation tetany | 45 years, male |

The questions provided in the menu (history-taking questions) came from the categories *main symptoms*, *prior history*, *allergies and medication*, *social and family history*, and *system review* by Bornemann (2016) and were evaluated in Fink et al (2021).

*History-taking Questions*

| Category | Code | Example |
| --- | --- | --- |
| Main symptoms | MS | Do you experience the complaints for the first time? |
| Prior history | PH | Do you know of any pre-existing conditions? |
| Allergies and medication | AM | Do you frequently have infections against which you take antibiotics? |
| Social and family history | SF | Have your parents or other relatives of your family passed away at a rather young age? |
| System review | SR | Has your weight changed within the last weeks? |

Bornemann, B. (2016). *Dokumentationsbögen der inneren Medizin und der Chirurgie für Anamnese und körperliche Untersuchung für die studentische Lehre in Deutschland*  (Diss., Institut für Didaktik und Ausbildungsforschung in der Medizin der Ludwig-Maximilians-Universität München). Retrieved from <https://edoc.ub.uni-muenchen.de/19166/>

Fink, M. C., Reitmeier, V., Stadler, M., Siebeck, M., Fischer, F., Fischer, M. R., . . . Fischer, M. R. (2021). Assessment of diagnostic competences with standardized patients versus virtual patients: Experimental study in the context of history taking. *Journal of Medical Internet Research*, *23*(3), e21196. <https://doi.org/10.2196/21196>

**Appendix S4: Reflection phases**

*Accompanying reflection*

| Nr | Question |
| --- | --- |
| 1. | Please name your current diagnosis. |
| 2. | What symptoms and findings agree with your current diagnosis? |
| 3. | What symptoms and findings disagree with your current diagnosis? |
| 4. | What questions will you ask the patient to examine your current diagnosis? |
| 5. | Please name alternative diagnosis in case your current diagnosis is incorrect. |
| 6. | What symptoms and findings agree with your alternative diagnoses? |
| 7. | What symptoms and findings disagree with your alternative diagnoses? |
| 8. | What questions will you ask the patient to examine your alternative diagnoses? |
| 9. | After reflecting on your current diagnosis and alternative diagnoses: What do you consider now the most probable diagnosis? |

*Concluding reflection*

| Nr | Question |
| --- | --- |
| 1. | Please name your current diagnosis. |
| 2. | What symptoms and findings agree with your current diagnosis? |
| 3. | What symptoms and findings disagree with your current diagnosis? |
| 4. | What questions you asked the patient were important to examine your current diagnosis? |
| 5. | Please name alternative diagnosis in case your current diagnosis is incorrect. |
| 6. | What symptoms and findings agree with your alternative diagnoses? |
| 7. | What symptoms and findings disagree with your alternative diagnoses? |
| 8. | What questions you asked the patient were important to examine your alternative diagnoses? |
| 9. | After reflecting on your current diagnosis and alternative diagnoses: What do you consider now the most probable diagnosis? |

Further information
Question 1, 5, and 9 used a long-menu format (see the section on diagnostic accuracy), all other questions free text input.

**Appendix S5: Manipulation checks**

Table 1 reports the duration participants spent working on the cases in different phases of the experiment and statistics from a one-way ANOVA comparing the groups. As expected, the duration of participants did not differ across groups in the pretest and posttest. As intended, participants in the experimental groups spent about four additional minutes on reflection.

Table 1

Duration Spent in the Conditions

| Phase | Concluding reflection | Accompanying reflection | Control group | *df* | *F* | *p* |
| --- | --- | --- | --- | --- | --- | --- |
| Pretest | 10.11 (1.81) | 10.27 (1.74) | 10.15 (1.56) | 2, 118 | 0.10 | .909 |
| Learning Phase | 13.71 (2.82) | 14.25 (2.13) | 8.97 (1.79) | 2, 117 | 64.01 | <.001 |
| Posttest | 8.71 (2.41) | 9.32 (2.07) | 9.31 (1.73) | 2, 118 | 1.14 | .322 |

*Note.* Means and (SDs) of the duration that participants spent in the conditions in minutes. One-way ANOVA results are reported across the three experimental conditions.

For another manipulation check, the number of words during reflection phases was calculated. Participants wrote in the accompanying reflection group on average *M* = 22.94, *SD* = 12.06 words per case during their reflections. Participants in the concluding reflection group wrote on average *M* = 30.11, *SD* = 21.04 words per case. There are two reasons that explain these relatively low word counts. First, participants summarized their reflections, as log-data inspection showed, primarily in bullet points. Second, participants spent during reflection only about half the time on free text questions and the other half on long-menu questions that did not contribute to the reported word count.
